# On the Role of Contact and System Stiffness in the Measurement of Principal Variables in Fretting Wear Testing

**DOI:** 10.3390/s20154152

**Published:** 2020-07-26

**Authors:** Diego Infante-García, Miguel Marco, Alaitz Zabala, Farshad Abbasi, Eugenio Giner, Iñigo Llavori

**Affiliations:** 1 Department of Mechanical Engineering, Universidad Carlos III de Madrid, Avda. de la Universidad 30, 28911 Leganés, Madrid, Spain; mimarcoe@ing.uc3m.es; 2 Surface Technologies, Mondragon University, Loramendi 4, 20500 Arrasate-Mondragon, Gipuzkoa, Spain; azabalae@mondragon.edu (A.Z.); illavori@mondragon.edu (I.L.); 3 Advanced Material Processes Research Group, Department of Mechanical and Industrial Production, Mondragon University, Loramendi 4, 20500 Arrasate-Mondragon, Gipuzkoa, Spain; fabbasi@mondragon.edu; 4 Centre of Research in Mechanical Engineering—CIIM, Department of Mechanical and Materials Engineering, Universitat Politècnica de València, Camino de Vera, 46022 Valencia, Spain; eginerm@mcm.upv.es

**Keywords:** fretting wear, tangential contact stiffness, numerical modelling

## Abstract

In this work, the role of the contact stiffness in the measurement of principal variables in fretting wear tests is assessed. Several fretting wear tribometers found in the literature, including one developed by the authors, are analysed and modelled using numerical methods. The results show the importance of the tribosystem stiffness and tangential contact stiffness in the displacement sensor calibration and in the correct numerical modelling of fretting wear tests, especially for flat-to-flat contact configuration. The study highlights that, in most cases, direct comparisons between fretting results with severe wear obtained with different tribometers cannot be performed if the contact stiffness is not properly considered during the development of the experiments.

## 1. Introduction

Fretting is related to at least two solid bodies in contact and subjected to oscillating forces which cause some microscopic relative tangential displacement. The magnitude of the relative tangential displacement usually ranges from 1 to 300 μm [1,2]. Fretting leads to two main surface damage processes along the contact area: wear of the surfaces and the initiation and propagation of fatigue cracks. In this way, fretting tests are usually classified in fretting fatigue and fretting wear tests.

**Figure 1:**
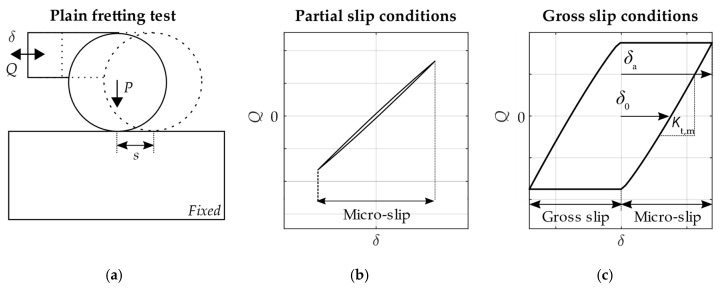
(**a**) Sketch of a plain fretting test; Schematic representation of frictional hysteresis loops obtained in a plain fretting test between elastic bodies with a cylinder to flat contact configuration using an Amontons-Coulomb friction model under (**b**) partial slip conditions and (**c**) gross slip conditions.

One of the most common tests found in the literature, usually named as fretting wear test or plain fretting test, is where an alternative tangential relative displacement *δ* is applied to a line contact between two bodies as sketched in Figure 1a. The macro relative tangential displacement *δ* is usually applied cyclically under constant amplitude in one of the bodies while keeping the other body fixed. If the relative macro displacement amplitude *δ*_a_ is not enough to cause slip *s* in all the contacting points, the contact is said to be under partial slip conditions (PSC). On the other hand, if all the contact area is sliding, the contact condition is said to be under gross slip conditions (GSC). The representation of the tangential load versus the relative tangential displacement during a fretting cycle gives the friction hysteresis cycle or, in other words, the fretting loop when related to fretting problems. Based on the well-known Cattaneo-Mindlin analysis [3,4], the narrow elliptical hysteresis loop shape is related with the PSC where the whole cycle is in micro-slip regime (see Figure 1b) [5]. On the contrary, the fretting loop shape in GSC shows a wider hysteresis loop, similar to a parallelogram shape (Figure 1c) [5]. Two distinct parts are observed in GSC: the micro-slip regime and the gross slip regime. The slope of the loop is related with the micro-slip regime where a significant part of the relative displacement is accommodated in the elastic deformation of the bodies in contact. On the other hand, the flat-topped part is associated with gross slip regime once the Coulomb limit is reached. During the gross slip regime, the tangential load is supposed to be independent of the displacement amplitude, and the relationship between the tangential load and the normal load is called the coefficient of friction *μ*_GP_. However, experimental fretting loops do not usually follow the idealized shape, specially under severe wear conditions, and the calculation is commonly not straightforward [6]. Several methods can be found in the literature to assess *μ*_GP_ [7].

In a fretting loop, the tangential contact stiffness is defined by the slope of the micro-slip regime. However, the micro-slip curve is theoretically non-linear as shown in its close form solution assuming elastic half-spaces and smooth surfaces [5,8]. Furthermore, other mechanisms that may affect the micro-slip region and tangential contact stiffness, such as the effect of the surface asperities, are usually hidden or difficult to assess when measuring the displacement between two points far away from the contact. This is caused by the large contribution of the displacement accommodated in the bulk deformation of the contacting bodies and system fixtures in the total displacement measured [9]. Nonetheless, the contact tangential stiffness is commonly calculated using curve linearization just after motion reversal, including the tangential stiffness belonging to the deformation of the bodies and the tribosystem.

The main variables in fretting tests are the normal contact force *P*, the tangential contact force *Q* and the micro-slip at the points in contact *s*. Strain gauges attached to proving rigs were traditionally employed in rotating bridge fretting tests to apply the contact load [2]. Nevertheless, force transducers in combination with different force actuators are more commonly employed for the application and measurement of the contact loads [10,11,12]. However, in specific applications such as steam generator tubes, fiber Fabry-Perot force sensors with reduced volume and high performance have been developed to assess the fretting damage [13,14]. In the literature, it is common to found screw-type mechanisms attached to springs [12,15,16], deadweight mechanisms [11,17,18,19,20] or servo-hydraulic systems [10,21,22] to apply a constant normal contact load. The fretting motion and tangential or bulk load are usually imposed using servo-hydraulic systems [16,22,23,24], mechanical linkages [15,19] or piezo-electric actuators [17,20] depending on the load requirements in terms of frequency and magnitude.

The micro-slip along the contact interface is considered the most difficult variable to measure *in-situ* in fretting testing. Thus, the slip amplitude *s*_a_ at each contact point is usually unknown during the experiments. Usually, the global or mean slip is usually extrapolated from the measured relative displacement measured *δ*. In most cases, *δ* is measured between two reference points relatively far away from the contact. In some cases, the measurement is performed between the specimen fixtures or directly with the displacement applied on the actuators. Sensors usually employed to measure the displacement are extensometers [12,25], linear variable differential transformers (LVDT) [11,21,26,27,28], capacitive position sensors [17] or laser displacement sensors [19]. Furthermore, the displacement sensors need to be carefully calibrated to consider the loads and compliance of the interference parts. On the other hand, recent several optical methods such as digital image correlation [29,30], fiber optic sensors [31] or laser interferometry [8,32,33] have allowed the relative tangential displacement measurement between two points close to the contact or to obtain the full field displacement of the contacting bodies. Therefore, the interference of the elastic displacement accommodated on the bodies in contact and fixtures is reduced. It should also be noted the required high precision in the measurement of the contact relative displacement, which is usually an order of magnitude below the micrometre.

The most common way to directly quantify the slip amplitude as a unique variable along the contact in a fretting test is by calculating the range of the displacement amplitude when the tangential load is zero *δ*_0_ (see Figure 1c) [5]. This variable gives us a mean value of the slip amplitude along the contact. An estimation of the full slip solution along the contact can be obtained using finite element analysis (FEA), but assuming significant simplifications. In the literature, finite element (FE) models usually consider part of the geometry of the contacting bodies, ignoring the fixtures and other elements to reduce the computational cost. In order to replicate the measured fretting loops, the applied boundary conditions, which usually neglect the rotation of the parts in contact, must consider the system and contact stiffness. Otherwise, the FE model would over constrain the motion of the bodies. Thus, the slip along the contact would not be correctly estimated.

In this work, fretting loops obtained by several fretting wear tribometers are analysed in order to estimate the system and contact stiffness and its influence in the correct test monitorization. In addition, a numerical analysis of the system stiffness influence on the wear modelling of fretting testing is presented using a FE numerical model. Furthermore, different hypothetical scenarios are analysed using a parametric FEA to highlight the importance of the correct measurement of the system stiffness and its impact on the principal fretting variables.

## 2. Materials and Methods

This section is organized as follows: In the first part, a description of several tribotesters is presented, which are broadly classified into two groups. The first presented tribotester, developed by the authors, is aimed to study an industrial application: the behaviour of thin steel wires under fretting conditions. The second group of presented tribometers belongs to several renowned research laboratories whose aim is the material damage quantification under plain fretting motion. In the second part, the numerical modelling of these tribometers is presented and a detailed description is given which is divided into two subsections. The first subsection corresponds to the fretting wear modelling of the thin steel wires fretting tests. In the second subsection, the description of a parametric finite element analysis of a plain fretting test is presented. Two hypothetical modelling scenarios are considered in fretting test with a cylinder to flat and a flat to flat with rounded edges contact configuration.

### 2.1. Fretting Tribotesters

#### 2.1.1. Fretting Tribosystem of Steel Wires

**Figure 2:**
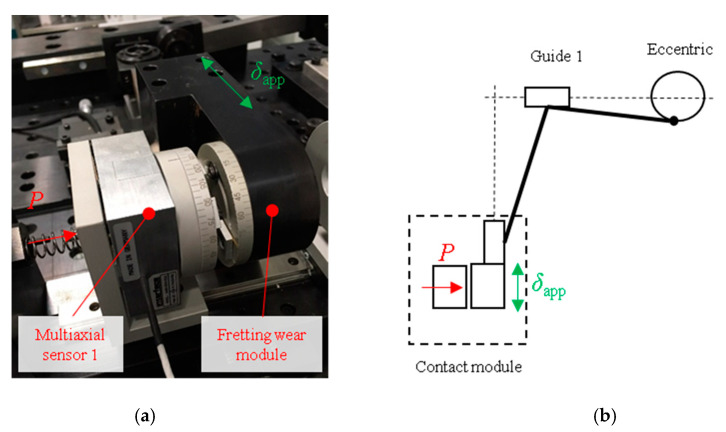
Fretting wear test apparatus: (**a**) Fretting wear contact module; (**b**) Schematic view of the fretting wear test configuration. Reproduced with permission of MDPI [15].

The steel wires fretting tribotester system shown in Figure 2 was designed and manufactured by the Surface Technologies Research Group from Mondragon University to perform fretting wear and fretting fatigue tests [15]. In the present study, the influence of the system stiffness on the fretting wear characterization was studied in detail. The test-rig employs a multi-axis sensor type 3A60 from Interface Inc. (Atlanta, GA, USA) to measure both the applied normal and tangential force directly. Traditionally, the normal and tangential force measurement sensors are decoupled, i.e., two uniaxial sensors are used separately in order to measure each component. However, this approach gives rise to a couple of disadvantages including linearity and crosstalk errors [15]. On the other hand, multiaxial sensors allow to have the same reference point for all the force components which is a key factor for measuring forces robustly in multiple directions.

A schematic view of the tribometer is shown in Figure 2. A contact force *P* is applied through a screw which is connected to a spring, in order to minimize any shock loading during the test. The spring stiffness is 1 N/mm and the normal force applied to the contact ranges between 1–10 N. Accordingly, the spring must be compressed 0.1 mm to cause a 0.1 N increase in the normal force. Note that diameters of steel wires in elevator ropes are usually below 0.5 mm and the range of wear depth in the worst case is lower than 40 µm. In addition, the displacement amplitude is applied through an eccentric mechanism connected to a DC motor. The eccentric is also connected to a mechanical system, which is capable of reducing the applied displacement by 2–3 orders of magnitude.

**Figure 3:**
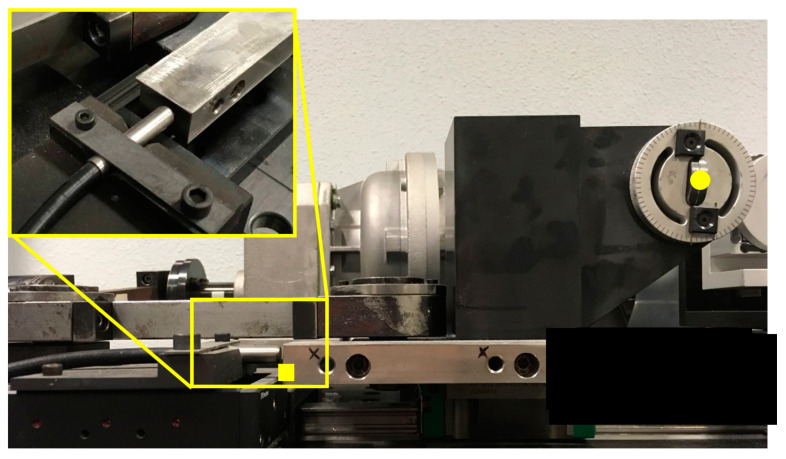
Detail of the displacement measurement module. Note that the displacement is measured remotely where the compliance of the contact system could influence the measurements.

Another important factor in a fretting wear test is the measurement of the displacement amplitude. For this purpose, the Tribometer employs an RC90 fiber-optic sensor (Philtec Inc., Annapolis, MD, USA) to measure the displacement accurately. As mentioned in the introduction section, the displacement at the contact point could not be measured easily; however, it can be estimated indirectly through the measurement of the displacement at the measurement module, which is captured remotely as shown in Figure 3 (solid yellow square symbol). It should be mentioned that, the actual displacement is generated on the location of the contact system as shown in Figure 3 (solid yellow circle symbol). Therefore, the accuracy of the remotely estimated actual displacement depends on the magnitude of both the applied load and compliance of the contact system. For small applied loads, the displacements of the two points are practically the same. However, under sufficiently large applied loads, the compliance of the contact system affects the estimation so that the displacements at measurement and displacement points shown in Figure 3 are not the same. In this particular case, the effect of contact compliance system should be taken into account. To this end, the two points of the tribometer are calibrated, so that the near displacement is known with remote measurement. This calibration was carried out by numerical simulation and validated by the displacement measured using a dial gauge indicator. Finally, a second order polynomial equation as a function of applied loads was developed.

#### 2.1.2. Fretting Wear Tribometers Using an Incomplete Contact Configuration

The main purpose of the following fretting tribosystems is to quantify the damage tolerance or strength in different pairs of materials under fretting conditions. To this end, the contact configuration employed in these tests is usually a cylinder or a sphere clamped on to a flat surface. Thus, the contact problem can be solved in closed form as explained in the introduction using the well-known Cattaneo-Mindlin analysis [3,4]. In all these tests, a pad is clamped to a flat specimen under constant normal load. Next, an alternative cyclic displacement under constant amplitude is applied in one of the bodies and the other body is kept fixed. In this section, the main characteristics and differences of each tribosystem are presented. The reader is referred to the original references for the corresponding full tribosystem description.

The first tribometer analysed in this work is the one used by Arnaud et al. [10]. They used a cyl.-to-flat (CTF) contact configuration, both specimens were made of a titanium alloy. Two hydraulic actuators were used, one responsible to apply the constant normal load and the other responsible to impose a constant cyclic relative tangential displacement. The displacement was recorded directly from the overall displacement imposed in the hydraulic actuator. Arnaud et al. [10] also tested a flat-to-flat with rounded edges configuration, which has a great interest because of its wide use in industrial applications. In addition, they observed some relative micro-rotation of the contacting bodies using digital image correlation during the tests, which led to contact misalignment. Another work belonging to the same research group is also analysed. In this case, Peteghem et al. [21] used a similar tribosystem configuration to the one employed by Arnaud et al. [10]. Contacting bodies were made of the same titanium alloy as in [10], although in this case the cylinder radius and the contact out of length are larger than the ones used in Arnaud et al. [10]. Another significant difference is that Peteghem et al. [21] monitored the tests to keep a constant *δ*_0_ instead of keeping constant *δ*_a_. as in [10].

Three works [11,18,28] from a research group at the University of Nottingham are also analysed. In these works, the same contact configuration and geometry is analysed, a cylinder with a diameter of 12 mm is clamped by dead weight on to a fixed flat specimen. A constant cyclic tangential displacement is applied to the cylinder using an electromagnetic vibrator. The displacement is recorded using a LVDT attached to the specimen holding block. In these works, the specimens are made of a titanium alloy and the main differences between the tests are the applied displacement amplitude [11,18] and the application of a coating to the specimens in one of the works [18].

The last analysed tribometer is the one developed by Ramesh and Gnanamoorthy [19], which uses an eccentric connected to an electric engine to impose the tangential displacement, similar to the tribometer of steel wires presented above. The authors employed a cyl.-to-cyl. (CTC) contact configuration. The normal contact load is applied using death weight between two crossed cylinders. Each cylinder is made of two hardened and tempered structural steels and a laser displacement sensor is used to measure the tangential displacement of the fixture.

**Figure 4:**
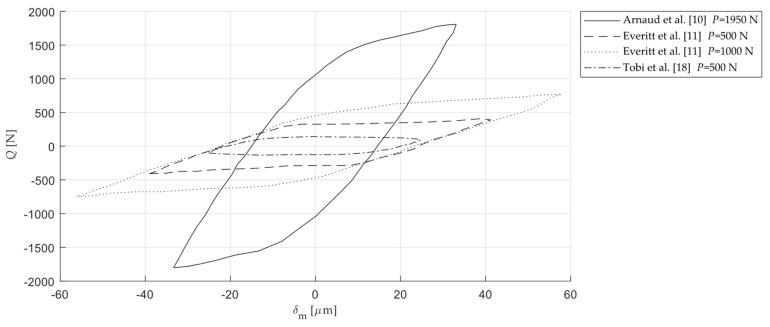
Frictional hysteresis loops in fretting wear tests of different works found in the literature [10,11,18] (reproduced with permission of Elsevier).

Fretting loops given by the works explained above are firstly digitalised as shown in Figure 4. Next, the fretting loops are analysed in terms of *δ*_0_, *δ*_a_, *K*_t,m_ and *μ*_max_. *K*_t,m_ is taken as the mean value calculated after linearization of the two micro-slip regimes just after reversal motion (half of the micro-slip region is considered in the linearization) and *μ*_max_ is calculated using the maximum value of the tangential force over a cycle. In some of the works, fretting loops are shown at different instants during the test, allowing to observe the evolution of the variables.

In order to obtain an estimation of the theoretical tangential contact stiffness *K*_t,c_ between the contacting bodies, a finite element analysis is performed for each fretting test with an in-line cyl.-to-cyl. contact configuration using a numerical model as the one presented in Section 2.2.2, but with the specimen geometry and loads adjusted to each test. *K*_t,c_ is calculated just after motion reversal between two points aligned to the centre of the contact and at a distance of 5 mm to the contact surface. For the crossed cyl.-to-cyl. contact configuration the Cattaneo-Mindlin solution is used to estimate *K*_t,c_ between two points located in the undeformed regions. The theoretical value of the tangential stiffness between two points located in the undeformed regions of two curved smooth surfaces assuming elastic half spaces is expressed as [3,4,34,35]:

(1) $$K_{t,c}=2G^{*}a$$

(2) $$\frac{1}{G^{*}}=\frac{2-\nu_{1}}{4G_{1}}+\frac{2-\nu_{2}}{4G_{2}}$$

being *a* the radius of the contact area and *v*_i_ and *G*_i_ the Poisson’s ratio and shear elastic modulus of each material in contact, respectively.

### 2.2. Numerical Modelling of Fretting Tests

#### 2.2.1. Steel Wires Fretting Numerical Modelling

**Figure 5:**
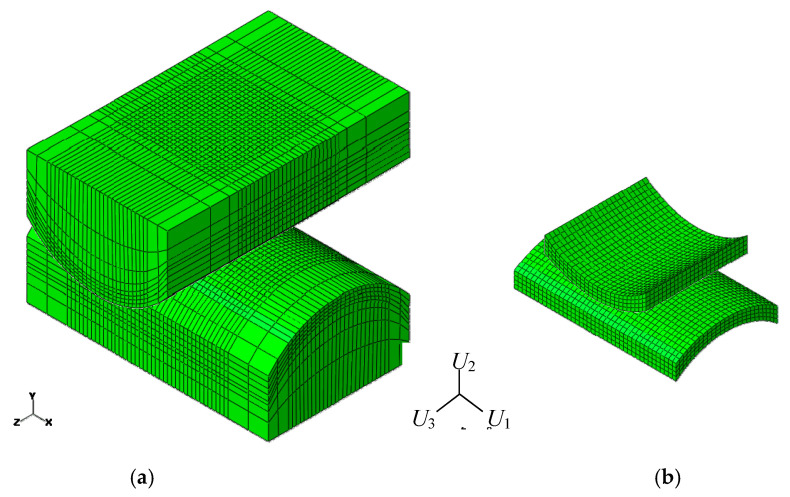
FE model consisting of C3D8 elements: (**a**) complete model; (**b**) closed-up view of the refined mesh at the contact region.

The crossed cylinder FE model was developed in Abaqus^©^ 6.14 as shown in Figure 5. Structural eight-node brick elements C3D8 were employed, with further mesh refinement at the contact region using the partitioning technique. The master-slave contact algorithm with the Lagrange multiplier method was used for the contact discretization in order to obtain an accurate resolution of the slip distribution, an aspect of great importance in fretting problems.

**Figure 6:**
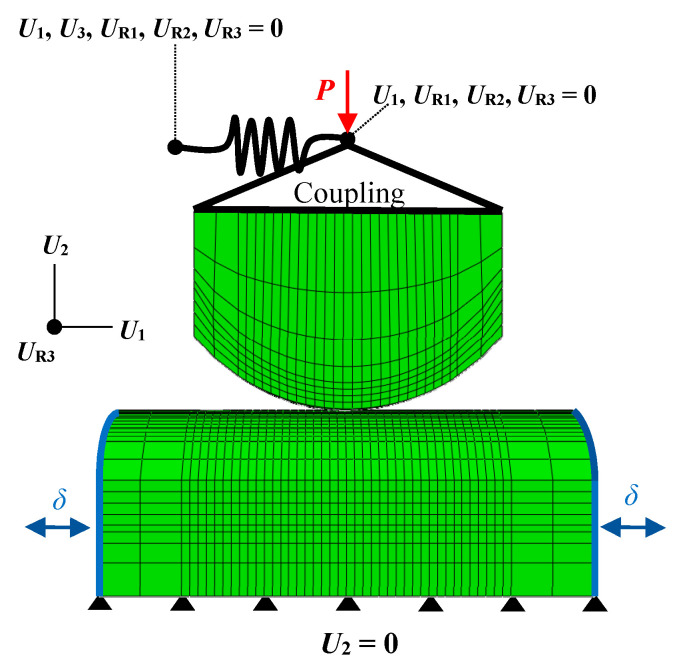
FE loading and boundary conditions in the 3D fretting steel wires model.

As shown in Figure 6, a contact force was applied to the upper wire by means of a kinematic coupling constraint so that the nodes were coupled to the rigid solid motion defined by a reference node. As a result, only the movement on the normal contact axis and parallel to the direction of motion was allowed (*U*_1_). The latter degree of freedom was applied in order to simulate the contact system stiffness, through a compliance spring attached to the upper wire as shown in Figure 6. On the lower wire, the fretting displacement amplitude was introduced according to the experimental measurements.

The wear simulation methodology employed in this study was similar to the one presented by Cruzado et al. [36] and the authors [37], where the energy wear equation was implemented in Abaqus FEA through the UMESHMOTION user subroutine. This subroutine has the capability of interactively updating the nodal positions during a FEA. The reader is referred to [37] for a full description of the wear model used in this work. The energy wear equation is defined as:

*V* = *α* ∑*E* (3)

where *V* is the wear volume, *α* is the energy wear coefficient and ∑*E* is the accumulated dissipated energy. The wear modelling consists of an iterative process in which the local energy equation is solved by means of both the shear traction and slip distributions obtained by the numerical simulation, as many times as required by the slip increments to complete the established number of cycles. Because this procedure has a high computational cost, a cycle jump technique is usually employed in order to accelerate the numerical simulation. Thus, the local energy equation is defined as:

Δ*h*(*x*,*t*) = Δ*n*·*α*·*q*(*x*,*t*)·Δ*s*(*x*,*t*) (4)

where Δ*h*(*x*,*t*), Δ*n*, *q*(*x*,*t*) and Δ*s*(*x*,*t*) represent the incremental wear depth, the cycle jump, the global energy wear coefficient, the shear traction and the relative slip at position *x* and time *t*, respectively. A variable coefficient of wear was introduced in order to replicate the experimental test. Finally, a friction coefficient of 0.62 was calculated from our experiments through the geometric independent coefficient of friction method. The reader is referred to [7] for a detailed explanation of the friction coefficient calculation.

#### 2.2.2. Finite Element Modelling of Fretting Tests Including Tribosystem Stiffness Influence

**Figure 7:**
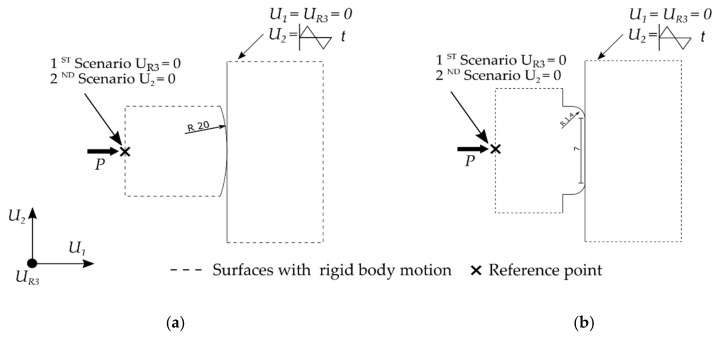
Schematic representation of the FE model with principal dimensions used for the numerical analysis with (**a**) flat-to-flat with rounded edges and (**b**) cyl.-to-flat contact configuration.

In this section, two bidimensional FE models of a classical fretting wear test have been developed using the FE commercial software Abaqus^©^. Two different line contact configurations are analysed: a flat-to-flat with rounded edges and a cyl.-to-flat contact configuration. The work of Arnaud et al. [10] is taken as a reference. Dimensions of flat specimens and pads given by Arnaud et al. [10] are fully modelled. Both interfaces between the pad and specimen with the fixtures are modelled as rigid bodies (see dashed lines in Figure 7). In all the numerical models, the specimen rigid surface translation is fixed in the contact normal direction *U*_1_ and rotation *U*_R3_, but a cyclic alternative motion in the tangential direction to the contact *U*_2_ is imposed with an amplitude of 35 µm. Several cycles are simulated until obtaining a frictional numerical shakedown.

Two hypothetical scenarios of the pad boundary conditions have been established in both contact configurations in order to introduce the stiffness of the tribosystem in the 2D FE models. Both scenarios represent two extreme modelling situations. The 1st scenario considers that the fixture is infinitely stiff in the out-of-plane rotation component *U*_R3_. In contrast, the 2nd scenario considers that the fixture is infinitely stiff in the tangential direction *U*_2_. Although a real tribosystem is not able to match these assumptions, both scenarios would allow us to analyse their individual effect on the principal variables in each contact configuration. In the 1st scenario, the pad rigid surface rotation is completely restricted. In addition, the rigid surface translation in the tangential direction is connected with a spring to ground *K*_t,s_. In the 2nd scenario, the pad rigid surface translation is fixed in the tangential direction *U*_2_ but allowing its rotation with respect to an axis represented with cross markers in Figure 7. Analogously to the 1st scenario, the rotational pad rigid surface motion is connected to a rotational spring *K*_θ,s_. Thus, the motion of the pad rigid surface is controlled in each scenario by the magnitude of the tangential *K*_t,s_ and rotational stiffnesses *K*_θ,s_ of the springs included in each scenario. In summary, the motion of the pad rigid surface in the 1st scenario is a translation along the *U*_2_ direction and in the 2nd scenario is a pure rotation along a perpendicular axis along the *U*_R3_ direction represented with a cross marker in Figure 7.

In all the finite element analyses corresponding to this subsection, linear 2D quadrilateral elements with plane strain formulation and reduced integration (CPE4R code in Abaqus^©^) were used in pad and specimen. To ensure an accurate prediction and to reduce the computational cost, a mesh sensitivity analysis was firstly performed, leading to an element size of 5 micrometres in the contact area and 1 mm far from the area of interest. A friction Coulomb model has been employed in the numerical model. The surface-to-surface formulation in combination with Lagrange multipliers has been employed for an accurate prediction of the shear traction distribution along the contact. Arnaud et al. [10] measured a friction coefficient at the sliding transition of 0.9. The effect of the variation of the friction coefficient on our numerical analyses is out of the scope of this paper. This way, a friction coefficient of 0.9 has been assumed in all the simulations. Pad and specimens are modelled as homogeneous isotropic elastic with a Young’s modulus of 116 GPa and a Poisson’s ratio of 0.3. The normal loads are 390 N/mm and 800 N/mm for the cyl.-to-flat and flat-to-flat with rounded edges, respectively.

## 3. Results

The results are organized in two sections as follows: In the first section, experimental fretting loops obtained using the steel wires fretting tribosystem are shown. The fretting loops are compared with the numerical predictions and the differences are discussed. In the second section, the experimental fretting loops of different tribosystems presented in the previous section of classical fretting tests are analysed. Lastly, a numerical analysis of the tribosystem stiffness influence in some of the principal fretting variables is presented assuming two modelling scenarios and two contact configurations.

### 3.1. Fretting Tribosystem of Steel Wires

#### 3.1.1. Experimental Fretting Loops for Comparison

Fretting wear tests in this study were carried out on wire of 0.45 mm diameter cold-drawn eutectoid carbon steel (0.8% C). A typical crossed cylinder (90°) contact configuration employed for all the tests. Experiments were conducted under a contact load of 2 N and an applied stroke of 2Δ = 120 μm under ambient environment. A fretting frequency of 3 Hz was applied for a duration of 100,000 cycles. Three repetition were performed to ensure repeatability. A summary of the test condition is presented in Table 1.

**Figure 8:**
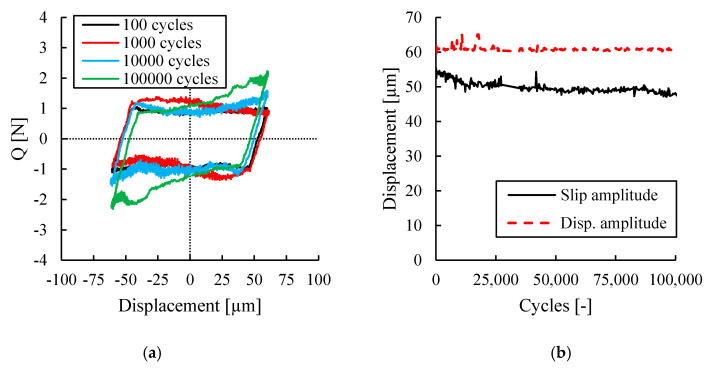
(**a**) Evolution of the fretting loop at different cycles; (**b**) Evolution of the slip and displacement amplitude during the test.

Fretting loops for one of the tests conducted at different cycles are presented in Figure 8. These fretting loops show a non-Coulomb fretting loop which is in a good agreement with author’s previous works [7,15] in which the tests were conducted under a combined fretting wear and fretting fatigue condition. Figure 8a shows that the fretting loop for 100 cycles is parallelogram in shape, much like the ideal fretting loop. However, due to the continuation of cycles, the fretting loop was transitioned so that the closed part of the loop was distorted after 10,000 cycles showing a non-Coulomb frictional behaviour. This transition demonstrated a gradual increase in dissipated energy in the contact interface accompanied by a simultaneous increase in wear debris loss from the contact zone and wear track geometry effects [7]. Additionally, the differences between the displacement amplitude *δ*_a_ and the slide amplitude *δ*_0_ are shown in Figure 8. In Figure 8b it can be observed that the displacement amplitude is almost constant throughout the test, with small variations due to outliers during the displacement measurement. On the other hand, it can be observed that the slip amplitude is always smaller than the displacement amplitude and it decreases with the continuation of cycles due to the non-Coulomb fretting loop behaviour. This behaviour is because the normal and friction force vectors rotate due to wear phenomenon, as shown by several authors [6,7,38].

#### 3.1.2. Numerical Results

**Figure 9:**
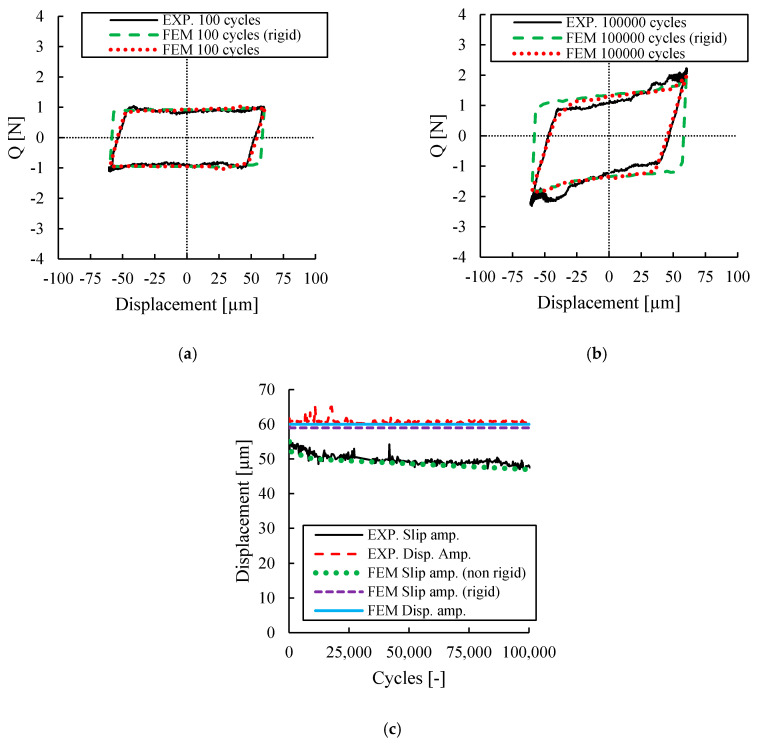
Comparison between the experimental and numerical results. Fretting loops: (**a**) 100 cycles; (**b**) 100,000 cycles. (**c**) Evolution of the slip and displacement amplitude for numerical model considering (non rigid) and not considering the stiffness of the contact system (rigid).

Figure 9 shows the experimental and numerical fretting loop for cycles 100 and 100,000, respectively. A typical flat topped-loop (Coulomb behaviour) can be observed at the fretting loop of 100 cycles shown in Figure 9a. However, as the fretting cycles increase, a more distorted loop is induced during the test, showing a non-Coulomb frictional behaviour as shown in Figure 9b. From Figure 9b it can also be seen that both the experimental and the numerical prediction results show a hysteresis cycle with the same distorted behaviour, having considered the compliance of the contact system through the use of a spring. From Figure 9a,b, it can be clearly seen that slip amplitude predicted by the numerical simulation is higher than the experimental measurement when the contact system is assumed to be completely rigid. On the other hand, as seen in Figure 9a,b, modelling the compliance of the test rig using spring elements results in an accurate prediction of the fretting loop measured by the experiments. The compliance of the spring introduced in the FE model is 167 N/mm. The compliance of the spring was calibrated so as to reproduce the fretting loops measured at the beginning of the test as shown in Figure 9a. With these results, it can be concluded that the compliance of the contact system is a dominant factor controlling the fretting response of the contact interface.

The comparison between the displacement amplitude and the slip amplitude predicted by the numerical simulations is shown in Figure 9c. It is seen that the numerical simulation considering the compliance of the contact system predicts accurately both the slip and displacement amplitude evolution. On the other hand, it can be observed that the numerical model assuming a rigid contact system is not able to capture the micro-slip part which arises due to the compliance of the contact system. These results reveal the importance of considering the real compliance of the contact system to predict accurately the contact response in fretting system. On the other hand, it can be also concluded that, although difficult, it is very important to have a very stiff contact system, so that the slip amplitude is always very close to the displacement amplitude (i.e., the latter is always a response of the system and difficult to control experimentally).

**Figure 10:**
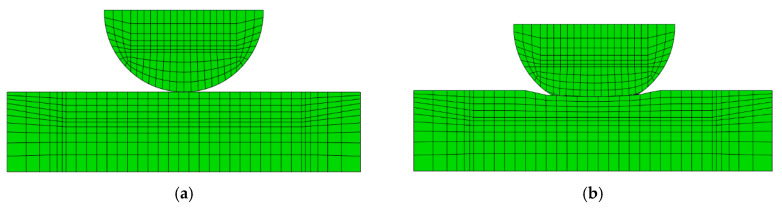
Detail of the contact zone (section on the axis of the applied displacement) for (**a**) fretting cycles of 100, where it is observed that the wear is negligible in the contact zone and (**b**) fretting cycles of 100,000, where it is observed that the wear is appreciable in the contact zone.

The transition in fretting loop reveals the effect of wear at the contact interface, which is intensified with an increasing number of fretting cycles. This point is more clearly seen in Figure 10a,b, which illustrate the numerical surface profile of the contact interface for fretting cycles of 100 and 100,000. Figure 10a clearly shows that the contact interface exhibits only small amount of wear for fretting cycles of 100, while for fretting cycles of 100,000 the wear track depth is higher and it becomes much shallower, as it is obvious in Figure 10b. This can explain why in Figure 9, the fretting loop gets wider at the closed part of the loop. Therefore, the 3D numerical simulation demonstrates that the distortion of the fretting loop is due to the increase in the size of the wear track throughout the number of cycles.

One of the advantages of numerical simulation is that it is possible to observe in detail the evolution of the wear footprint and associate it with the evolution of the fretting loop, something that cannot be done easily in the experimental test.

### 3.2. Analysis of Fretting Tribosystem and Contact Stiffness

#### 3.2.1. Tangential Contact Stiffness Analysis of Different Tribosystem

**Figure 11:**
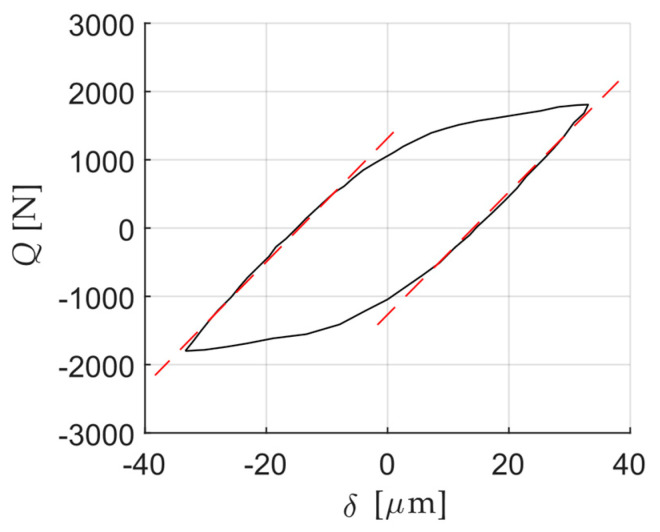
Fretting loop (black continuous line) and curve linearization of both micro-slip regimes (dashed red line) for the fretting loop of Arnaud et al. [10].

Figure 11 shows an example of the curve linearization performed to obtain the slope of the micro-slip and hence the measured contact tangential stiffness. The same procedure has been applied for all the fretting loops analysed in this work. Table 2 summarizes the fretting loops belonging to the works presented in Section 2.1.2. As expected, the measured tangential stiffness is lower than the estimated theoretical values. The variation of the measured tangential stiffness ranges from −37% to −94%. The principal reason might be that in all these tribometers the relative displacement is remotely measured, at a distance larger than the distance employed in the calculation of the theoretical value. Thus, the compliance of the tribosystem hinders the measurements. It should be mentioned that useful information that could aid the interpretation of the results is not reported in the published data, such as the position of the reference point where the displacement is measured or the sensor calibration. In addition, other mechanisms may affect the measured contact tangential stiffness which are not included in our theoretical estimations of the tangential stiffness such as the surface roughness [23].

The contact tangential stiffness directly affects the estimated slip amplitude, assumed as the relative displacement amplitude when the tangential load is zero *δ*_0_. As can be observed in Table 2, approximately 50% of the applied displacement is accommodated in the elastic deformation of the contacting bodies or fixtures. Furthermore, if the applied displacement amplitude is kept constant and the tangential forces increase during a test due to wear, the estimated global slip amplitude *δ*_0_ decreases as observed in the fretting loops belonging to references [11,18,19,28]. This reduction is more significant at low slip values with high compliance fixing systems. However, the wear surface evolution may also affect the micro-slip along the contact as well as the tangential contact stiffness. As a direct consequence, it is not possible to stablish direct comparisons between tribosystems or tests with different compliance. This result is also applied to tests peformed in the same tribosystem, but using a material with significant different elastic properties. On the other hand, this phenomenon is avoided if the global slip amplitude is kept constant during a test such as in reference [21]. Note that two fretting loops here analysed employ coated bodies in contact [18]. Lastly, we note that the effect of coating has been included solely in the theoretical calculation of the tangential stiffness through the reduction of the friction coefficient to a value of 0.3 in the 2D FE model, as reported in [18].

#### 3.2.2. Parametric Analysis of the Tribosystem Stiffness Influence on Contact Variables

First of all, the relationships between the tangential stiffness related with the tribosystem *K*_t,s_ and the tangential stiffness measured in the resulting fretting loop just after the reversal motion *K*_t,m_ have been established. The measured displacement *δ* is calculated between ground and a specimen node located on the rigid surface. Note that all the nodes located on the specimen rigid surface of the model describe the same path. Next, four cases have been analysed for each of the two scenarios presented above. For each contact configuration, *K*_t,s_ and *K*_θ,s_ are defined using the relationships established previously in such a way that the *K*_t,m_ calculated in the fretting loop is identical for each case in both scenarios. Table 3 and Table 4 show the values of the tangential stiffness measured in the fretting loops obtained in our cases for each contact configuration, respectively. Furthermore, the values of the tangential stiffnesses modelled for each case, modelling scenario and contact configuration are also given in both tables. In addition, the maximum angle of rotation (*θ*_max_) estimated by FE in the pad rigid surface at maximum tangential load obtained for the second scenario is also given for each contact configuration. Note that stiffnesses in Table 3 and Table 4 are given per unit length.

**Figure 12:**
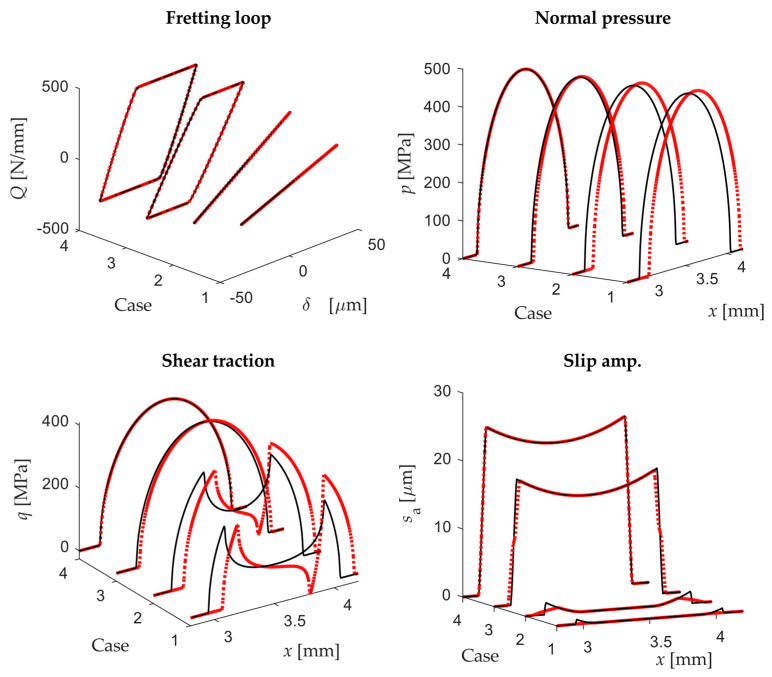
Fretting loops, normal and shear tractions at maximum displacement and slip amplitude along the contact for the four cases analysed with the 1st scenario (black continuous line) and 2nd scenario (red dotted line) using a cyl.-to-flat contact configuration.

Figure 12 shows the main contact variables of the four cases analysed using the cyl.-to-flat contact configuration: the fretting loop, the contact pressure and shear traction at the instant of maximum displacement and the slip amplitude along the contact during a cycle. As can be observed, the fretting loops obtained by both scenarios are exactly the same (both plots overlap in Figure 12), being half of them under PSC (1 and 2) and the other half under GSC (3 and 4). In addition, no significant differences were found for the rest of variables. It is noticeable that for a low rotational stiffness in the second scenario, the pad rolls over the flat specimen, reaching more distant points at the specimen surface than for the same case in the first scenario. In this way, the contacting points on the edges are changing and so the slip amplitude slightly decreases on the edges. In the 2nd scenario, the maximum angle of rotation measured in the pad rigid surface at maximum tangential load was 0.17° for case 1. This result shows the high sensitivity of the problem to the rotational system stiffness.

**Figure 13:**
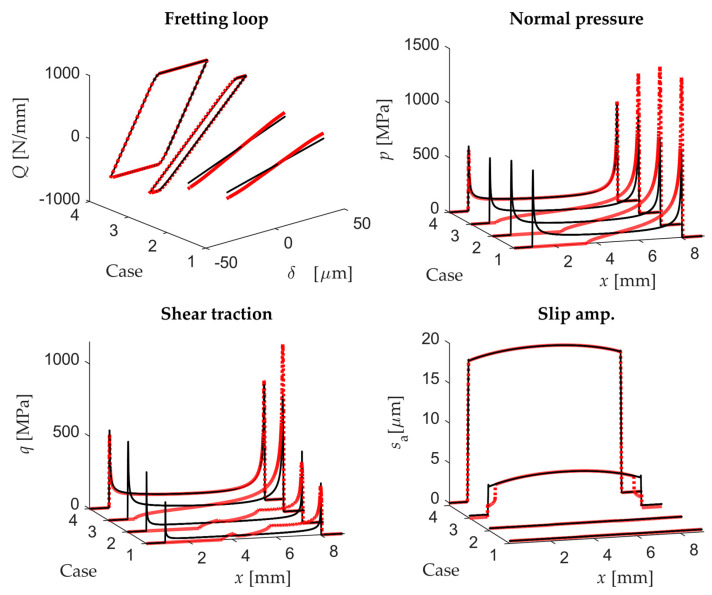
Fretting loops, normal and shear tractions at maximum displacement and slip amplitude along the contact for the four cases analysed with the 1st scenario (black continuous line) and 2nd scenario (red dotted line) using a flat-to-flat contact configuration.

Analogously, Figure 13 shows the main contact variables of the four cases analysed but using the flat-to-flat with rounded edges contact configuration. As shown, a slightly different fretting loop is observed for both scenarios under PSC, although they have identical tangential stiffness after motion reversal. On the other hand, even although the fretting loop is identical under GSC, quite significant changes are observed in the contact pressure and shear traction distribution along the contact as shown in Figure 13. The misalignment produced by the pad rotation for the second scenario causes a reduction of the contact area and promotes the stress concentration at the contact edges. In addition, the slip amplitude along the contact decreases significantly on both sides under GSC when there is lifting on one of the sides. Similar to the cyl.-to-flat contact configuration, the maximum angle of rotation at maximum tangential load was 0.18° for case 1. This result shows again the high sensitivity of the problem to the micro-rotation of the bodies.

**Figure 14:**
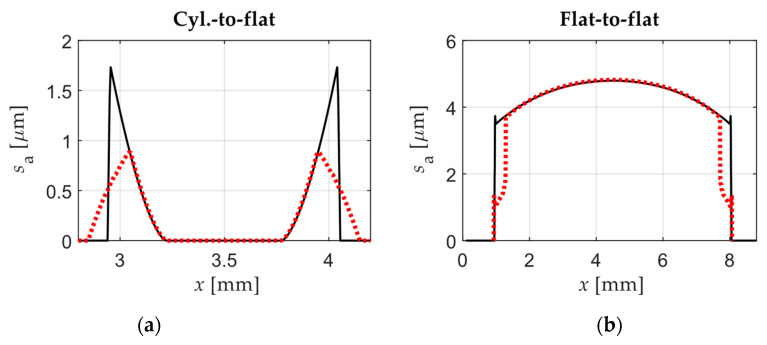
(**a**) Slip amplitude along the contact in case 2 with cyl.-to-flat contact for the 1st scenario (black continuous line) and 2nd scenario (red dotted line); (**b**) Slip amplitude along the contact in case 3 with flat-to-flat contact for the 1st scenario (black continuous line) and 2nd scenario (red dotted line).

In both contact configurations, significant differences were found particularly in fretting loops closed to the transition point from PSR to GSR. For instance, the slip amplitude along the contact under PSR for the cyl.-to-flat contact configuration decreased in the second scenario as shown in Figure 14a.

On the other hand, in case 3 using a flat-to-flat contact configuration under GSR the slip distribution slightly increased in the middle contact area and decreased on the edges (see Figure 14b). However, in the same case the normal pressure and shear traction drastically changed when comparing both scenarios. As expected, and as a general rule, the flat-to-flat contact is more sensitive to micro-rotations due to the misalignment of the flat surfaces.

## 4. Conclusions

In this work, a critical analysis of the contact and tribosystem stiffness influence on the measurement of principal variables in fretting wear tests has been performed. The analysis combines experimental results with numerical simulations. Several fretting wear tribometers have been reviewed, including one developed by the authors, and analysed in order to assess the system and contact tangential stiffness. The main conclusion is that direct comparisons between results obtained in fretting wear tests with a different tangential contact stiffness cannot be generally performed. The tangential stiffness must be considered in the calibration of the sensors and the fretting motion actuator control units. In this way, system stiffness plays a fundamental role in the correct measurement of the principal variables as shown by the numerical analyses. An accurate measurement of the main fretting variables is necessary to establish proper damage relationships. In addition, numerical models should be equivalent to the whole system stiffness and the necessary values need to be experimentally measured. The system stiffness measurement should consider the micro-rotations of the fixtures, especially when using a flat-to-flat contact configurations as shown in the parametric FEA.

## Figures and Tables

**Table 1:** Fretting tribological test conditions.

Properties	Symbol	Unit	Value	Tribosystem
Contact load	*P*	N	2	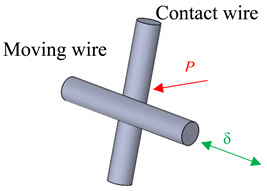
Crossing angle	*α*	°	90	
Average contact pressure	*p*_av_	MPa	3100	
Maximum Hertz pressure	*p*_max_	MPa	4650	
Tangential stress ratio	*R*_Q_	-	−1	
Stroke	2*δ*_a_	μm	120	
Frequency	*υ*	Hz	3	
Number of cycles	*N*	-	100 × 10^3^	
Lubricant		-	None	
Temperature	*T*	°C	22 ± 2	
Atmosphere		-	Laboratory air	
Relative humidity	RH	%	50 ± 5	

**Table 2:** Summary of the total tangential stiffness of different fretting tests found in the literature.

Ref.	Contact Conf.	Pad/Spec. Material	*R* [mm]	*P* [N]	*a* [mm]	*μ*_max_	*δ*_a_ [μm]	*δ*_0_ [μm]	*N* [cycles]	*K*_t,m_ [N/μm]	*K*_t,c_^1^ [N/μm]	*(K*_t,c_ − *K*_t,m_)/*K*_t,c_ [%]
[10]	CTF	Ti-6Al-4V	40	1950	0.549	0.93	33.22	14.12	-	88.24	139.10	-
[11]	CTF	Ti-6Al-4V	6	500	0.076	0.82	40.15	22.67	5.00 × 10^3^	22.48	161.70	−86%
[11]	CTF	Ti-6Al-4V	6	1000	0.108	0.77	56.87	22.98	5.00 × 10^3^	21.83	170.70	−87%
[11]	CTF	Ti-6Al-4V	6	500	0.076	0.96	40.37	20.41	1.00 × 10^5^	21.84	161.70	−86%
[11]	CTF	Ti-6Al-4V	6	1000	0.108	0.91	60.06	19.54	1.00 × 10^5^	21.08	170.70	−88%
[21]	CTF	Ti-6Al-4V	80	8523	1.286	0.59	134.89	77.17	2.50 × 10^2^	85.58	289.20	−70%
[21]	CTF	Ti-6Al-4V	80	8523	1.286	0.68	141.31	71.13	3.00 × 10^3^	79.48	289.20	−73%
[18]	CTF	Ti-6Al-4V (coated)	6	500	0.076	0.28	24.94	20.26	1.00 × 10^5^	18.62	161.70	−88%
[18]	CTF	Ti-6Al-4V (coated)	6	500	0.076	0.44	24.00	13.67	6.00 × 10^5^	18.38	161.70	−89%
[28]	CTF	Ti-6Al-4V	6	500	0.076	0.88	24.72	7.87	5.00 × 10^3^	24.16	161.70	−85%
[28]	CTF	Ti-6Al-4V	6	500	0.076	0.97	24.96	5.32	5.00 × 10^4^	23.76	161.70	−85%
[19]	CTC	En31/En24	5	19.6	0.087	0.51	25.69	15.01	1.00 × 10^3^	1.19	16.03 *	−93%
[19]	CTC	En31/En24	5	19.6	0.087	0.54	27.56	13.24	9.90 × 10^4^	0.90	16.03 *	−94%

^1^ The estimated tangential contact stiffness *K***_t,c_** is calculated using FEA between two equidistant points to the centre of contact at a distance of 5 mm for the in-line CTF contact configuration and using the Cattaneo-Mindlin solution calculated between two points located in the undeformed regions for the CTC (crossed) contact configuration (*).

**Table 3:** Summary of the four cases analysed in first and second scenario using a cyl.-to-flat contact configuration.

Case	*K*_t,m_ [N/mm^2^]	1st Scenario	2nd Scenario	
		*K*_t,s_ [N/mm^2^]	*K*_θ,s_ [N/rad]	*θ*_max_ [°]
1	4.35 × 10^3^	5.00 × 10^3^	5.28 × 10^5^	1.67 × 10^−1^
2	7.64 × 10^3^	1.00 × 10^4^	1.06 × 10^6^	1.45 × 10^−1^
3	1.94 × 10^4^	5.00 × 10^4^	5.30 × 10^6^	3.89 × 10^−2^
4	2.99 × 10^4^	5.00 × 10^5^	5.64 × 10^7^	3.67 × 10^−3^

**Table 4:** Summary of the four cases analysed in first and second scenario using a flat-to-flat with rounded edges contact configuration.

Case	*K*_t,m_ [N/mm^2^]	1st Scenario	2nd Scenario	
		*K*_t,s_ [N/mm^2^]	*K*_θ,s_ [N/rad]	*θ*_max_ [°]
1	6.10 × 10^3^	7.00 × 10^3^	3.17 × 10^5^	1.80 × 10^−1^
2	8.94 × 10^3^	1.10 × 10^4^	6.37 × 10^5^	1.71 × 10^−1^
3	2.34 × 10^4^	4.66 × 10^4^	3.28 × 10^6^	9.51 × 10^−2^
4	4.35 × 10^4^	5.84 × 10^5^	4.82 × 10^7^	7.70 × 10^−3^
